# Data on the axial response of steel tubes infilled with rubberised alkali-activated concrete

**DOI:** 10.1016/j.dib.2024.110172

**Published:** 2024-02-08

**Authors:** Mohamed Elzeadani, Dan V. Bompa, Ahmed Y. Elghazouli

**Affiliations:** a Department of Civil and Environmental Engineering, Imperial College London, London SW7 2BU, UK; b Department of Civil and Environmental Engineering, University of Surrey, Guildford GU2 7XH, UK; c Department of Civil and Environmental Engineering, Hong Kong Polytechnic University, Hong Kong

**Keywords:** Concrete-filled steel tubes, Rubberised concrete, Axial load-shortening response, Finite element modelling

## Abstract

The presented data cover experimental and numerical axial load-shortening results of steel tubes infilled with rubberised alkali-activated concrete. The experimental data are obtained from 36 concrete filled steel tube specimens with circular and square cross-sections, length-to-diameter/width ratios of 2 and 4, and three different rubber contents in the concrete infill. The data from the numerical assessment cover the axial load-shortening response of over 300 finite element models. These cover a wide range of concrete infill strengths and rubber contents, steel tube grades, specimen widths, and steel tube wall thicknesses. Detailed descriptions of the material and methods, experimental testing, and numerical modelling procedures are also provided. The data reported herein supports the discussion in the research article “Axial compressive behaviour of composite steel elements incorporating rubberised alkali-activated concrete,” and in the case of the numerical parametric assessment, give for the first time the full axial load-shortening response of all the models considered.

Specifications Table

**Table:** 

Subject	Civil and Structural Engineering.
Specific subject area	Axial response of steel tubes infilled with rubberised concrete.
Data format	Raw, Analysed.
Type of data	Table, Figure, Finite element model input files.
Data collection	Experimental tests were performed on physical specimens. The experimental data was then used to validate the numerical models built in ABAQUS/CAE. A parametric assessment covering 315 finite element models was then performed.
Data source location	Department of Civil and Environmental Engineering, Imperial College London, SW7 2BU London, United Kingdom.
Data accessibility	Repository name: Mendeley Data Data identification number: https://doi.org/10.17632/5g6s4g8yjn.1 Direct URL to data: https://data.mendeley.com/datasets/5g6s4g8yjn/1
Related research article	M. Elzeadani, D. V. Bompa, A. Y. Elghazouli. (2024). Axial compressive behaviour of composite steel elements incorporating rubberised alkali-activated concrete. Journal of Constructional Steel Research, 212, 108276. https://doi.org/10.1016/j.jcsr.2023.108276.

## 1 Value of the Data

• The experimental data can serve as a benchmark for validating future simulation models concerned with the axial response of steel tubes infilled with rubberised alkali-activated concrete.
• The simulation methods and data presented can help researchers calibrate and refine their models to ensure they accurately represent the physical tests.
• The parametric simulation data permit the investigation of several design parameters and their effect on the overall axial response, which is valuable for the development of comprehensive and robust design expressions.

## 2 Background

The concrete in axially loaded concrete-filled steel tubes is subjected to passive confinement which affects its response. This confinement is well understood for conventional concrete materials, but not so for novel concrete including rubberised alkali-activated concrete [1]. The present article reports the axial load-shortening data obtained from experimental and numerical investigations of steel tubes infilled with rubberised alkali-activated concrete. The data highlight the effect of various parameters on the overall response and support the discussion in the original research article [2]. The presented data also provide for the first time the full details of the axial load-shortening response of all the 315 models investigated in the numerical study, which is useful for characterising the influence of the studied parameters on aspects such as stiffness, ductility, and energy dissipation.

## 3 Data Description

The data cover the experimental and numerical simulation results of steel tubes infilled with rubberised alkali-activated concrete tested under concentric axial compression. The experimental tests include specimens with circular and square cross-sectional shapes, two length-to-diameter (*L/D*) or length-to-width (*L/B*) ratios of 2 and 4, and three volumetric crumb rubber replacement ratios of total natural aggregates (i.e., 0%, 30%, and 60% corresponding to concrete mixes R00, R30 and R60, respectively).

**Fig. 1:**
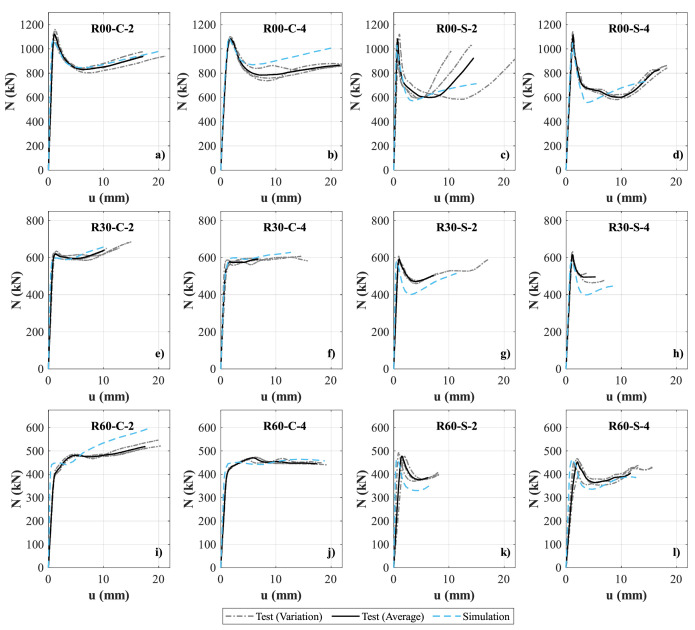
Test and simulated axial load-shortening curves: a) R00-C-2; b) R00-C-4; c) R00-S-2; d) R00-S-4; e) R30-C-2; f) R30-C-4; g) R30-S-2; h) R30-S-4; i) R60-C-2; j) R60-C-4; k) R60-S-2; l) R60-S-4.

The data repository is divided into two folders: 1) Experimental dataset, and 2) Numerical dataset. The experimental dataset folder contains three data files, covering i) concrete material properties, ii) steel material properties, and iii) the experimental axial load-shortening results (i.e., *N-u* response). The experimental results are given in excel spreadsheets and include the raw data from the experiments and the calculated average values. The concrete material properties file includes the stress–strain curves of the three concrete mixes considered, i.e., R00, R30 and R60. The steel material properties file includes the stress-strain curves of tensile coupon tests taken from the circular hollow sections (CHS), and the flat and corner sides of the square hollow sections (SHS). The experimental axial load-shortening results file contains the response of 12 different specimens, as shown in Fig. 1. The naming format of these specimens is taken as Rxx-C/S-n, where Rxx is the infill concrete mix (i.e., R00, R30 or R60), C/S refers to the cross-section shape (i.e., C for circular and S for square), and n refers to the *L/D* or *L/B* ratio (i.e., 2 or 4).

The numerical dataset folder contains three subfolders named: i) Abaqus Input Files, ii) Input material properties, and iii) Numerical results. The first subfolder contains the ABAQUS/CAE finite element modelling input files of the twelve main models used for validation purposes. The input material properties subfolder contains three excel spreadsheets named: i) steel input material properties, ii) concrete input material properties – circular sections, and iii) concrete input material properties – square sections. These excel spreadsheets give the full steel and concrete input material properties used for the numerical assessment in ABAQUS.

The numerical results subfolder contains four excel spreadsheets named: i) numerical validation results, ii) parametric assessment results – circular specimens, iii) parametric assessment results – square specimens, and iv) summary of the numerical axial strength results. The numerical validation results file contains the axial load-shortening simulation results of the 12 physical specimens, and are also shown in Fig. 1. The parametric assessment results files cover the axial load-shortening response of 315 models covering elements with different reference concrete strengths (50, 70, and 90 MPa), varying volumetric rubber replacement ratios of total natural aggregates (0%, 15%, 30%, 45%, and 60%), different steel yield strengths (235, 355, and 460 MPa), circular and square cross-sections; 100- and 150-mm outer steel tube diameter/width; and 3- and 5-mm steel tube thickness. All models considered in the parametric assessment had a *L/D* or *L/B* ratio of 2 and did not exceed the section slenderness limits given in Eurocode 4 [3] and AISC 360 [4] to ensure the attainment of the full yield strength of the steel tube prior to the initiation of local buckling. Lastly, the summary of the numerical axial strength results file contains the axial strength of the 315 models covered in the parametric assessment. In cases where post-peak hardening was observed in the load-shortening response with no clear peak, the peak axial strength, *N_u_*, was taken as that corresponding to 2% axial displacement of the overall specimen length. A PDF file called parametric assessment plots was also added to the Numerical results subfolder, and this gives plots of the axial load-shortening curves of the 315 models considered in the parametric assessment.

The naming format adopted in the numerical dataset files is C/S-Cxx-Ryy-Yzzz-D/t, where C/S refers to the cross-section shape (i.e., C for circular and S for square), Cxx refers to the reference non-rubberised concrete grade (i.e., C50, C70, C90), Ryy refers to the concrete infill type (i.e., R00, R15, R30, R45, and R60), Yzzz refers to the steel tube grade (i.e., Y235, Y355, Y460), D refers to the outer diameter/width (i.e., 100, 150 mm), and t refers to the steel tube thickness (i.e., 3, 5 mm). Therefore, the name C-C70-R45-Y460-100/5 refers to a circular specimen with a reference non-rubberised concrete strength of 70 MPa, a rubber replacement ratio of total natural aggregates (*ρ_vr_*) of 45%, a steel tube yield strength of 460 MPa, an outer diameter of 100 mm, and a steel tube thickness of 5 mm.

Besides the experimental and numerical dataset folders, the source publication file [2], published as open access in Journal of Constructional Steel Research, is also uploaded in the dataset repository.

## 4 Experimental Design, Materials and Methods

### 4.1 Specimens’ preparation and material properties

The binder used to form alkali-activated concrete was a mixture of ground granulated blast furnace slag (GGBS), fly ash (FA), and solid sodium metasilicate anhydrous (Na_2_SiO_3_). Sodium tetraborate decahydrate-borax (Na_2_B_4_O_7_·10H_2_O), in a solid state, was used as a chemical admixture. The fine and coarse natural aggregates used were river sand (0–5 mm) and crushed gravel (5–10 mm) with a specific gravity of 2.67 and 2.69, respectively. Crumb rubber particles with six different sizes, i.e., 0–0.5 mm, 0.5–0.8 mm, 1.0–2.5 mm, 2–4 mm, 4–10 mm, and 10–20 mm, and a specific gravity of 0.97, were used to replace 0, 30, and 60% of the total natural aggregates by volume, corresponding to mixes R00, R30 and R60, respectively. Details of the concrete mix designs are given in Table 1 and further details on the concrete mix design selection can be found in Elzeadani et al. [5].

**Table 1:** Concrete mix designs.

Constituent	R00	R30	R60
GGBS (kg/m^3^)	480	480	480
FA (kg/m^3^)	120	120	120
Na_2_SiO_3_ (kg/m^3^)	72	72	72
Borax (kg/m^3^)	30	30	30
Fine aggregates (0–5 mm) (kg/m^3^)	675	472.5	270
Coarse aggregates (5–10 mm) (kg/m^3^)	825	577.5	330
Crumb rubber (0–0.5 mm) (kg/m^3^)	0	8.1	16.3
Crumb rubber (0.5–0.8 mm) (kg/m^3^)	0	8.1	16.3
Crumb rubber (1–2.5 mm) (kg/m^3^)	0	24.4	48.8
Crumb rubber (2–4 mm) (kg/m^3^)	0	32.6	65.1
Crumb rubber (4–10 mm) (kg/m^3^)	0	16.3	32.6
Crumb rubber (10–20 mm) (kg/m^3^)	0	73.3	146.5
Water (L/m^3^)	180	180	180

The concrete was prepared by first mixing all the dry ingredients for 5–8 min. Water was then added, and mixing was continued for another 5–8 min. The fresh concrete was subsequently poured in the steel tubes in three layers and each layer was compacted on a vibrating table for 30–60 s. Specimens were allowed to cure in ambient conditions and testing was performed after 28 days of curing.

**Fig. 2:**
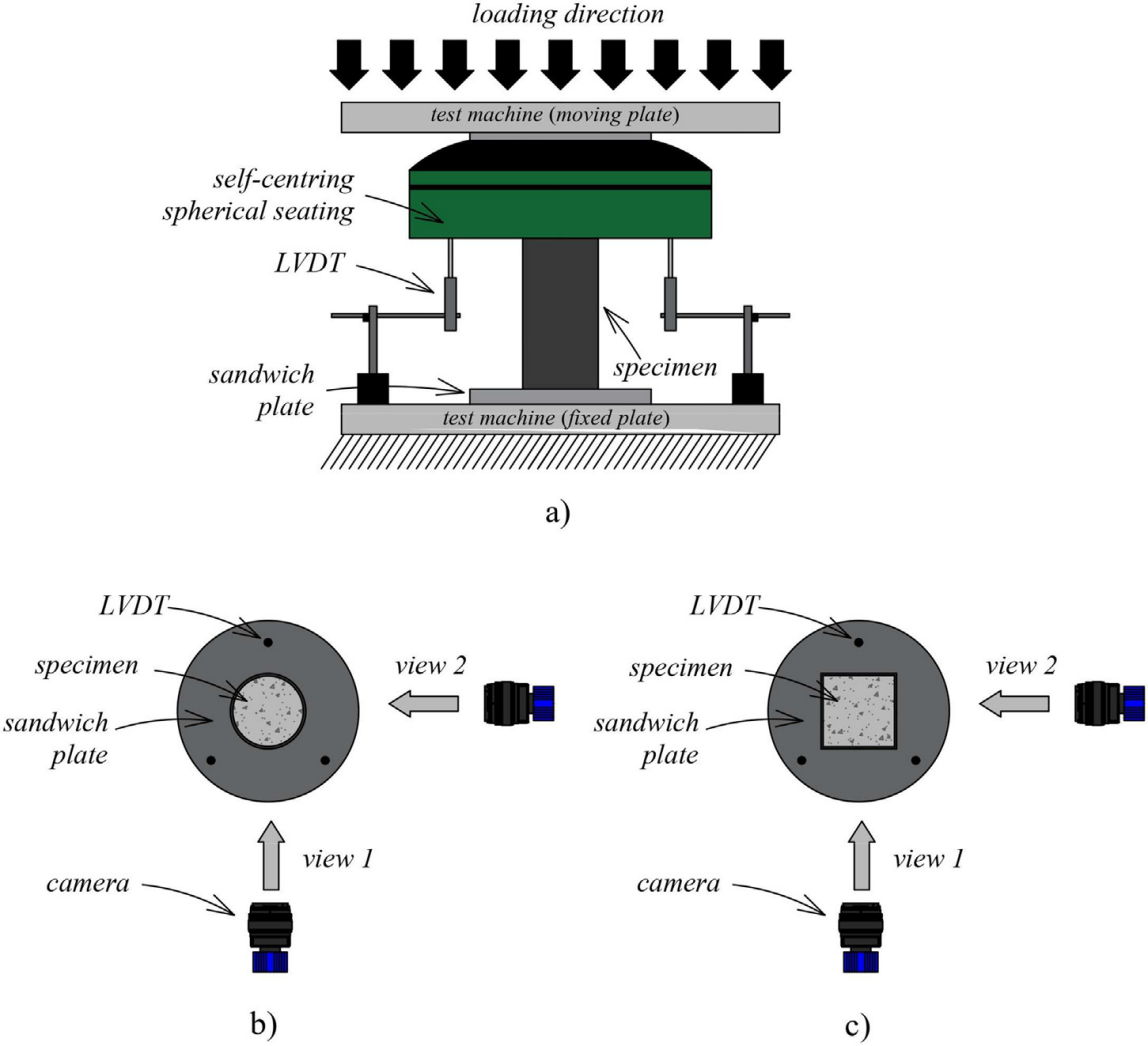
Test setup: a) layout; b) plan view of circular specimens; c) plan view of square specimens.

The concrete material properties are given in Table 2 and include the concrete hardened density, the 28-day cylinder compressive strength (*f_c_*), the axial and lateral crushing strains corresponding to *f_c_* on the stress-strain response (*ε_cr,1_* and *ε_cr,2_*, respectively), and the concrete Young's modulus (*E_c_*). The unconfined mechanical properties of concrete were determined by testing three nominally identical Ø100 × 200 mm cylinders in compression in an Instron Satec 3500 machine. The compression tests were performed in displacement control at a rate of 0.25 mm/min. A 4-camera digital image correlation (DIC) system, similar to that shown in Fig. 2 and described further in the following sub-section, was used to monitor the axial and lateral displacements in the concrete specimens, from which *ε_cr,1_* and *ε_cr,2_* were determined. The full stress-strain response of the unconfined concrete can be found in the related research article [2].

**Table 2:** Concrete and steel material properties.

Concrete	Density	*f_c_*	*ε_cr,1_*	*ε_cr,2_*	*E_c_*
	(kg/m^3^)	(MPa)	(‰)	(‰)	(GPa)
R00	2319	71.8	2.66	−1.23	30.7
R30	2112	22.1	2.09	−2.24	12.7
R60	1877	8.7	1.97	−2.26	6.0

Steel	Section	*f_y_*	*E_s_*	*f_u_*	*ε_u_*
		(MPa)	(GPa)	(MPa)	(%)

CHS	Circular	311.1	203.1	415.2	11.5
SHS	Square	361.6	201.0	419.9	8.9

The steel circular hollow sections (CHS) had a measured outer diameter, *D*, and thickness, *t*, of 102 and 3.4 mm, respectively. The steel square hollow section (SHS) had a measured outer width, *B*, of 100 mm, a thickness, *t*, of 2.8 mm, and inner and outer corner radii of 4 and 6.5 mm, respectively. The material properties of the CHS and SHS are also given in Table 2 and include the yield stress (*f_y_*), the steel Young's modulus (*E_s_*), ultimate strength (*f_u_*), and ultimate strain (*ε_u_*). The steel properties were determined by testing five nominally identical standard steel coupons in uniaxial tension using an Instron Satec 250 kN machine. The tensile tests were performed in displacement control at a rate of 0.42 mm/min in the pre-yield regime and 1.50 mm/min in the post yield regime in line with ISO 6892-1 [6]. For the SHS, and due to the development of residual stresses during the steel working process, the material properties were determined by testing coupons taken from the flat and corner regions of the section (i.e., five coupons in each case). The average SHS properties, which are shown in Table 2, have been determined by averaging the properties from the flat and corner coupons in proportion to their area in the section. Further details on the steel coupon tests, including the full tensile stress-strain curves, can be found in the related research article [2].

### 4.2 Experimental testing

Three nominally identical specimens were tested for a specific cross-section shape (i.e., circular, square), *L/D* or *L/B* ratio (i.e., 2, 4), and concrete infill (i.e., R00, R30, R60), giving a total of 36 concrete-filled steel tube (CFST) specimens. All specimens were tested for axial compression as illustrated in Fig. 2 in an Instron Satec 3500 kN machine. Specimens were placed on the bottom platform of the testing machine, and loads were applied using a spherical seating with a 3D rotating hinge that was attached to the loading platen of the Instron machine. The spherical seating was used to minimise any loading eccentricities on the tested specimens. The tests were conducted in displacement control at a rate of 0.25 mm/min and 1 mm/min in the pre-peak and post-peak regimes, respectively.

Specimen displacements were monitored using a 4-camera digital image correlation (DIC) system at a rate of 1 Hz (i.e., 1 image per second). Two cameras were used to monitor one face of the specimens while another two cameras were used to monitor an adjacent face as shown in Fig. 2. To allow the DIC system to track pixel displacements in the images, the specimens were speckled with white dots before testing to form a contrasting surface. Images were post-processed in LaVision DaVis 10.2 software [7]. Virtual extensometers were then placed along the length of the specimens, which were used to extract the displacement results. For each specimen, displacements from two sides facing the cameras were extracted, which were then averaged to obtain the specimen displacement. Three linear variable differential transducers (LVDTs), spaced equally around the specimens, were used as secondary measurement instruments to monitor the overall displacement. It should be noted that the displacements shown in this article, as well as those given in the data repository, were all determined from the DIC system. Further details on the experimental testing arrangement can be found in the related research article [2].

### 4.3 Modelling procedures

The numerical assessment was carried out in ABAQUS/CAE [8]. The steel tube and core concrete were modelled using four-node shell elements with reduced integration (S4R) and eight-node solid elements with reduced integration (C3D8R), respectively. A mesh sensitivity study, performed on R00-C-2 and R00-S-2, showed that a mesh size of up to 15 mm gave good predictions, while a mesh coarser than 20 mm led to a drop in the axial capacity. As such, a mesh size of 10- and 15-mm were used in the validation of the modelling procedures and parametric assessments, respectively. A larger mesh size was used in the latter mainly to reduce computation time given the large number of parameters considered. “Sweep” and “structured” mesh types were used for the steel and concrete, respectively.

**Fig. 3:**
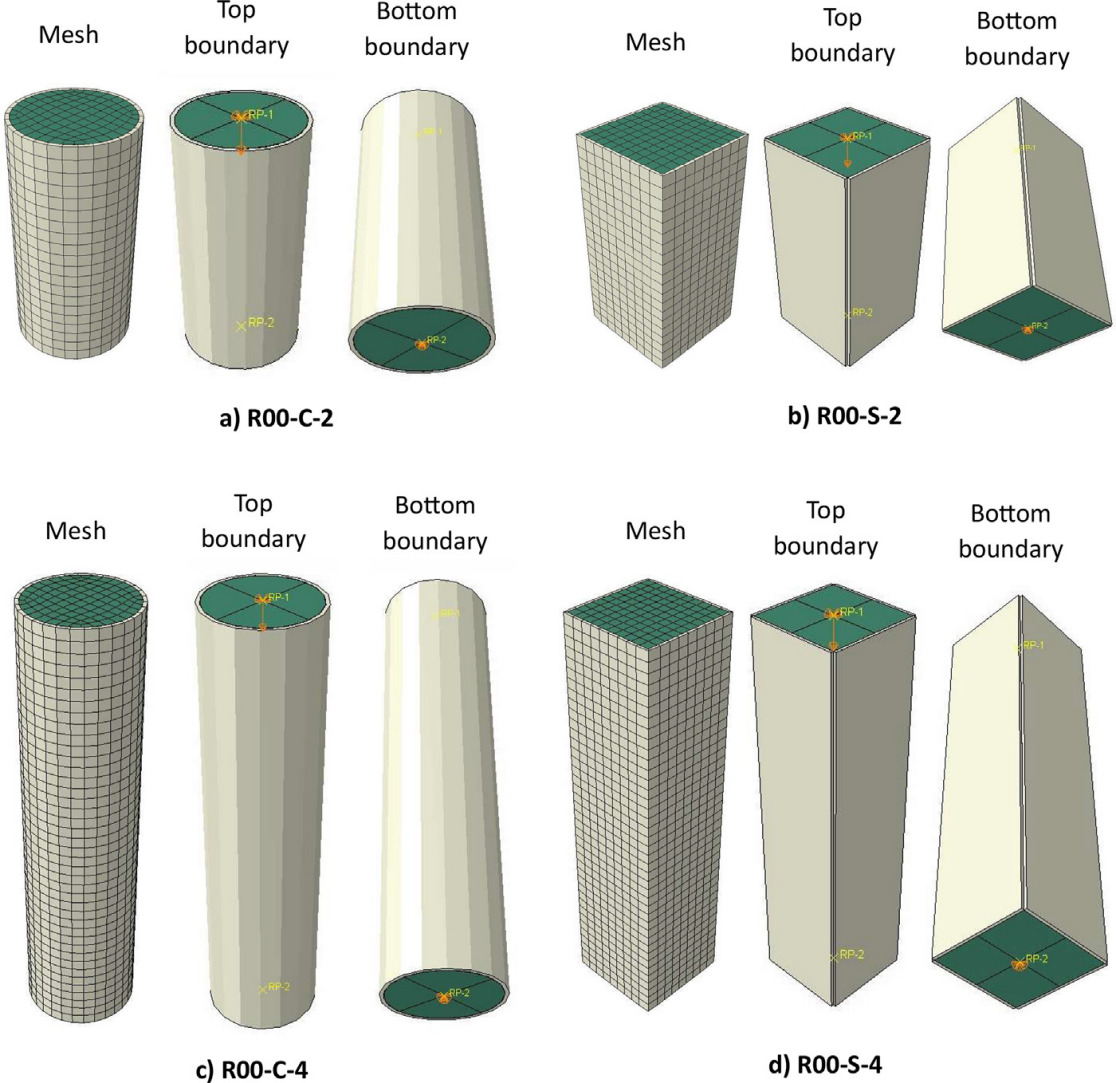
Finite element models: a) R00-C-S; b) R00-S-2; c) R00-C-4; d) R00-S-4.

A “surface-to-surface contact” between the inner steel tube and outer concrete surface was assigned. The inner steel tube surface was defined as the master surface while the outer concrete core surface was defined as the slave surface. “Hard contact” between the surfaces was specified in the normal direction, which prohibits penetration in compression but allows separation in tension. A “penalty” property with a 0.5 friction coefficient was specified for the tangential behaviour. The top and bottom surfaces of the steel tube and concrete core were tied to a reference point at the centre of each surface using the “rigid body” constraint. Translational degrees of freedom were restrained at both ends except for the vertical displacement at the loaded end, while the rotational degrees of freedom were released at both ends. The load was applied by assigning a displacement to the top reference point. Fig. 3 shows the finite element validation models for the R00 infilled specimens, illustrating the mesh density, and applied boundary conditions together with the position of the reference points.

The steel constitutive material model adopted is given by Eqs. (1)–(5), where *σ* is the engineering stress, *ε* is the engineering strain, *ε_y_* is the yield strain, *ε_p_* is the strain corresponding to the start of strain-hardening, *E_p_* is the strain-hardening slope taken as 0.02*E_s_*, and *p* is a strain-hardening parameter. ABAQUS requires converting the engineering stress (*σ*) and engineering strain (*ε*) to true stress (*σ_true_*) and true strain (*ε_true_*) values, which are given by Eqs. (6) and (7), respectively. The elastic and plastic behaviour of the steel tube were defined using the “isotropic” material type. The steel Young's modulus, *E_s_*, was taken as 200 GPa, while the steel Poisson's ratio, *v_s_*, was taken as 0.3. The inelastic strain (*ε_in_*), used to define the plastic behaviour, was determined following Eq. (8).

(1) $$\sigma =\left\{ \begin{array}{cc} E_{s}\varepsilon & 0\leq \varepsilon_{s}<\varepsilon_{y}\\ f_{y} & \varepsilon_{y}\leq \varepsilon <\varepsilon_{p}\\ f_{u}-\left(f_{u}-f_{y}\right){\left(\frac{\varepsilon_{u}-\varepsilon }{\varepsilon_{u}-\varepsilon_{p}}\right)}^{p} & \varepsilon_{p}\leq \varepsilon <\varepsilon_{u}\\ f_{u} & \varepsilon \geq \varepsilon_{u} \end{array}\right.$$

(2) $$p=\;E_{p}\left(\frac{\varepsilon_{u}-\varepsilon_{p}}{f_{u}-f_{y}}\right)$$

(3) $$\varepsilon_{p}=\left\{ \begin{array}{cc} 15\varepsilon_{y} & f_{y}\leq 300\;MPa\\ \left[15-0.018\left(f_{y}-300\right)\right]\varepsilon_{y} & \;300\;MPa<0\leq 800\;MPa \end{array}\right.$$

(4) $$\varepsilon_{u}=\left\{ \begin{array}{cc} 100\varepsilon_{y} & \;f_{y}\leq 300\;MPa\\ \left[100-0.15\left(f_{y}-300\right)\right]\varepsilon_{y} & 300\;MPa<0\leq 800\;MPa \end{array}\right.$$

(5) $$f_{u}=\left\{ \begin{array}{cc} \left[1.6-2\;\times 10^{-3}\left(f_{y}-200\right)\right]f_{y}\; & \;200\;MPa\leq \;f_{y}\leq 400\;MPa\\ \left[1.2-3.75\;\times 10^{-4}\left(f_{y}-400\right)\right]f_{y}\; & 400\;MPa<f\_y\leq 800\;MPa \end{array}\right.$$

(6) $$\sigma_{true}=\;\sigma \left(1+\varepsilon \right)$$

(7) $$\varepsilon_{true}=\ln\left(1+\varepsilon \right)$$

(8) $$\varepsilon_{in}=\varepsilon_{true}-\;\frac{\sigma_{true}}{E_{s}}$$

The constitutive model used for the concrete infill is given by Eqs. (9)–(23), where *ε_c,el_* is the strain corresponding to the end of the perfectly elastic region, *f_c,2_* is the confined concrete compressive strength, *f_c0_* is the reference non-rubberised concrete strength, *ε_cr,1,0_* is the reference non-rubberised concrete axial crushing strain, *f_r_* is the residual concrete strength, *ρ_vr_* is the volumetric rubber replacement ratio of the total natural aggregates, *α* is a parameter that controls the shape of the descending branch and depends on the confinement ratio, *ζ_c_*. An “isotropic” material type was assigned for the elastic concrete behaviour. The concrete Young's modulus, *E_c_*, is given by Eq. (24) while the concrete Poisson's ratio, *v_c_*, is given by Eq. (25). The variables *V_c_* and *V_r_* in Eq. (25) refer to the volumetric proportions of concrete and rubber in the concrete matrix, respectively, while *v_c0_* and *v_r_* refer to the Poisson's ratio of the reference non-rubberised concrete and rubber, respectively, taken as 0.2 and 0.5, each.

(9) $$\sigma =\;E_{c}\varepsilon \;\to \;\varepsilon \;\leq \;\varepsilon_{c,el}=0.3f_{c,2}/E_{c}$$

(10) $$\frac{\sigma }{f_{c,2}}=\left[\frac{5}{3}\eta_{1}-\eta_{1}^{2}+0.3\right]\;\to \;\varepsilon_{c,el}<\;\varepsilon \leq \;\varepsilon_{cr,1}$$

(11) $$\sigma =f_{c,2}\;\to \;\varepsilon_{cr,1}<\;\varepsilon \leq \;\varepsilon_{cr,1,2}\;\left(circular\;CFST\right)$$

(12) $$\sigma =\;\left\{ \begin{array}{cc} \;f_{r}+\left(f_{c,2}-f_{r}\right)exp\left[-{\left(\frac{\varepsilon -\varepsilon_{cr,1,2}}{\alpha }\right)}^{0.92}\right]\; & \varepsilon \geq \varepsilon_{cr,1,2}\;\left(circular\;CFST\right)\\ f_{r}+\left(f_{c,2}-f_{r}\right)exp\left[-{\left(\frac{\varepsilon -\varepsilon_{cr,1}}{\alpha }\right)}^{0.92}\right]\; & \varepsilon \geq \varepsilon_{cr,1}\;\left(square\;CFST\right) \end{array}\right.$$

(13a) $$\frac{f_{c}}{f_{c0}}=\;\frac{1}{1+2{\left(\frac{3\lambda \rho_{vr}}{2}\right)}^{3/2}}$$

(13b) $$\lambda =\left\{ \begin{array}{c} 2.9\;\to replacement\;of\;FNA\;and\;CNA\\ 2.43\;\to replacement\;of\;FNA\;\left(\leq 5mm\right)\\ 2.08\;\to replacement\;of\;CNA\;(>5mm) \end{array}\right.$$

(14) $$f_{c,2}=\left\{ \begin{array}{c} 1.5f_{c}\to circular\;CFST\\ 1.0f_{c}\to square\;CFST \end{array}\right.$$

(15) $$\eta_{1}=\;\frac{\left(\varepsilon -\;\varepsilon_{c,el}\right)}{\varepsilon_{cr,1}}$$

(16) $$\varepsilon_{cr,1,0}=0.8\;\times {f_{c,2}}^{0.31}\times 10^{-3}$$

(17) $$\varepsilon_{cr,1}=\varepsilon_{cr,1,0}\;\times \;{\left(1-\;\rho_{vr}\right)}^{1/4}$$

(18) $$\frac{\varepsilon_{cr,1,2}}{\varepsilon_{cr,1}}=\;e^{k}$$

(19) $$k=\;\left(2.9224-0.00367f_{c}\right){\left(\frac{f_{B}}{f_{c}}\right)}^{0.3124+0.002f_{c}}$$

(20) $$f_{B}=\;\frac{\left(1+0.027f_{y}\right)e^{-0.02\frac{D}{t}}}{1+1.6e^{-10}{\left(f_{c}\right)}^{4.8}}\;\left(circular\;CFST\right)$$

(21) $$f_{r}=\;\left\{ \begin{array}{c} 0.2f_{c}\;\left(circular\;CFST\right)\\ 0.1f_{c}\;\left(square\;CFST\right) \end{array}\right.$$

(22) $$\alpha =\;\left\{ \begin{array}{cc} 0.04-\;\frac{0.036}{1+e^{6.08\xi_{c}-3.49}} & \left(circular\;CFST\right)\\ 0.008+0.0005\xi_{c}\; & \left(square\;CFST\right) \end{array}\right.$$

(23) $$\xi_{c}=\;\frac{A_{s}f_{y}}{A_{c}f_{c,2}}$$

(24) $$E_{c}=\;\left\{ \begin{array}{c} 7200{\left(\frac{f_{c}}{10}\right)}^{2/3}\to \;\rho_{vr}=0\\ 6500{\left(\frac{f_{c}}{10}\right)}^{2/3}\to \;\rho_{vr}>0 \end{array}\right.$$

(25) $$v_{c}=\;\frac{v_{c0}V_{c}+\;v_{r}V_{r}}{V_{c}+\;V_{r}}$$

The concrete plastic behaviour was defined using the concrete damaged plasticity (CDP) model in ABAQUS. The material properties for the CDP model are given by Eqs. (26)–(29) and include the ratio of the biaxial-to-uniaxially loaded concrete strength (*f_b0_/f_c_*), the ratio of the tensile meridian's second stress invariant to that of the compressive meridian's second stress invariant (*K_c_*), and dilation angle (*ψ*). The flow potential eccentricity (*e*) and viscosity parameter needed for the CDP model were taken as 0.1 and 10^−6^, respectively. The concrete inelastic strain, *ε_in_*, required for the definition of the compressive behaviour in the CDP model is given by Eq. (30). The concrete compressive damage parameter, *d_c_*, was taken as that given by Eq. (31). As for the concrete tensile behaviour, a linear relationship was assumed up to the direct tensile strength, *f_t_*, given by Eq. (32). The tensile softening behaviour was characterised by the fracture energy, *G_f_*, given by Eq. (33), where *d_max_* is the maximum natural aggregate size taken as 10 mm in this study.

(26) $$\frac{f_{b0}}{f_{c}}=1.5{\left(f_{c}\right)}^{-0.075}$$

(27) $$K_{c}=\;\frac{5.5}{5+2{\left(f_{c}\right)}^{0.075}}$$

(28) $$\psi =\;\left\{ \begin{array}{c} \psi_{0}\left(1-\rho_{vr}\right)\;\left(circular\;CFST\right)\\ 40\left(1-\rho_{vr}\right)\;\left(square\;CFST\right) \end{array}\right.$$

(29) $$\psi_{0}=\left\{ \begin{array}{c} 56.3\left(1-\xi_{c}\right)\;\xi_{c}\leq 0.5\\ 6.672e^{\frac{7.4}{4.64+\xi_{c}}}\;\xi_{c}>0.5 \end{array}\right.$$

(30) $$\varepsilon_{in}=\;\varepsilon -\frac{\sigma }{E_{c}}$$

(31) $$d_{c}=1-\frac{\sigma }{f_{c,2}}$$

(32) $$f_{t}=0.26f_{c}^{2/3}$$

(33) $$G_{f}=\left(0.0469{d_{max}}^{2}\;-0.5d_{max}+26\right){\left(\frac{f_{c}}{10}\right)}^{0.7}$$

## Limitations

The experimental and numerical axial load-shortening data presented here covered non-slender circular and square concrete-filled steel tubes infilled with rubberised alkali-activated concrete and subjected to concentric axial load. The material properties considered covered core reference non-rubberised concrete strengths between 50 and 90 MPa, up to 60% volumetric crumb rubber replacement of total natural aggregates in concrete, and steel tube yield strengths between 235 and 460 MPa. The specimens in the experimental and numerical studies were chosen to ensure the development of the full yield strength in the steel tubes prior to the initiation of local buckling.

## Ethics Statement

The current work does not involve human subjects, animal experiments, or any data collected from social media platforms.

## CRediT authorship contribution statement

**Mohamed Elzeadani:** Conceptualization, Methodology, Data curation, Formal analysis, Software, Writing – original draft. **Dan V. Bompa:** Conceptualization, Methodology, Project administration, Supervision, Writing – review & editing. **Ahmed Y. Elghazouli:** Conceptualization, Methodology, Funding acquisition, Project administration, Supervision, Writing – review & editing.

## Data Availability

Axial response of steel tubes infilled with rubberised alkali-activated concrete (Original data) (Mendeley Data).
